# Computational Discovery of Selective Carbonic Anhydrase IX (CA IX) Inhibitors via Pharmacophore Modeling and Molecular Simulations for Cancer Therapy

**DOI:** 10.3390/ijms26178465

**Published:** 2025-08-30

**Authors:** Nahlah Makki Almansour

**Affiliations:** Department of Biology, College of Science, University of Hafr Al Batin, Hafr Al Batin 31991, Saudi Arabia; nahlama@uhb.edu.sa

**Keywords:** carbonic anhydrase IX, inhibitors, pharmacophore model, molecular docking

## Abstract

Carbonic anhydrase IX (CA IX) is a transmembrane metalloenzyme that is increased in tumor cells under hypoxia and plays an important role in solid tumor acidification. It is a marker of tumor hypoxia and a prognostic factor in human malignancies. Given the critical role of CA IX and their over expression in many cancer tissues, they have emerged as a promising target for developing novel anticancer therapeutics. In this study we designed a pharmacophore model based on known inhibitors to screen small compound libraries to discover potential inhibitors of CA IX. Molecular docking experiments discovered that four compounds ZINC613262012, ZINC427910039, ZINC616453231, and DB00482 exhibited a strong binding affinity towards CA IX, mimicking the interaction pattern similar to native inhibitors. Molecular dynamics simulations and an MM-PBSA analysis revealed ZINC613262012, ZINC427910039, and DB00482 as the most potential and stable inhibitors with the binding free energies −10.92, −18.77, and −12.29 kcal/mol, respectively. In addition, DFT-based analyses supported their favorable electronic properties, further validating their potential as CA IX inhibitors. These three hits demonstrated a greater stability and compactness relative to the known inhibitors, suggesting these might be used CA IX inhibitors to treat tumors.

## 1. Introduction

Hypoxia is a hallmark of solid tumors, which are highly prevalent and associated with high mortality rates [1]. Solid tumors account for approximately 90% of all cancers originating from a single mutated cell and metastasize to essential organs like the liver, lungs, and brain. Tumor progression is marked by rapid cell growth and significant changes to the tumor cell microenvironment, primarily due to insufficient oxygen supply, resulting in hypoxia or even anoxia [2,3]. Hypoxia arises due to the rapid proliferation of tumor cells, which often outpaces the supply of oxygen, activating HIF-1α (hypoxia inducible factor-1α), which regulates genes that aid tumor survival, including carbonic anhydrase IX (CA IX), thereby reducing the effectiveness of anticancer treatments [4,5,6,7]. Carbonic anhydrase enzymes are crucial components of pH regulation, essential for the survival of cancer cells. CA IX expression is closely linked to the upregulation of HIF-related elements and has a significant role in promoting tumor cell survival, proliferation, migration, adhesion, pH regulation, and cell signaling. Its restricted expression in normal tissues and its location on the external membrane of tumor cells make CA IX an appealing target for therapeutic intervention in cancer treatment [8,9,10,11,12]. Although CA IX expression is minimal in healthy tissues, it is significantly upregulated in hypoxia-induced tumors, where it plays a critical role in maintaining pH and ion balance [13]. Targeting CA IX with inhibitors such as sulfonamides, coumarins, and dithiocarbamates blocks its catalytic activity, disrupting pH regulation, and hindering tumor growth, which makes CA IX a promising target for anticancer therapy [14,15,16]. Inhibiting human CA IX (hCA IX) disrupts the crucial hypoxic extracellular environment that tumor cells rely on for survival, invasion, and metastasis, thereby impairing their ability to thrive under these conditions. Carbonic anhydrases (CAs) are evolutionary, conserved metalloenzymes that catalyze the reversible conversion of CO_2_ to bicarbonate (HCO_3_) and H^+^, which is essential for physiological and pathological processes such as pH homeostasis [17].

**Figure 1:**
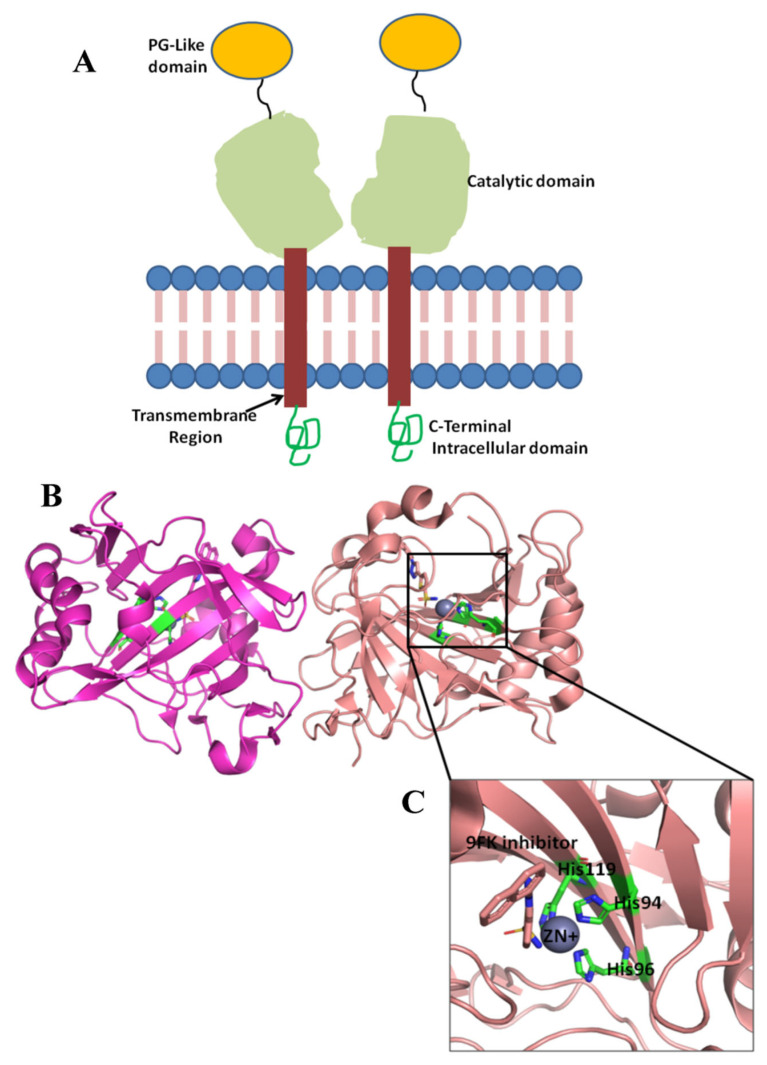
(**A**) Schematic representation of the domains of the CA IX. Proteoglycan (PG)-like domain, catalytic domain (CD), transmembrane region (TM), and C-Terminal Intracellular domain (IC). (**B**) Catalytic domain dimer (PDB ID 5FL4), (**C**) inhibitor 9FK bound in the active site, and three histidine residues (His 94, 96, and 116) coordinating Zn^2+^ ion.

CA IX is a 459 amino acid transmembrane protein which is encoded by the CA9 gene. This protein exists in dimer form. The structure consists of several key domains: a proteoglycan-like domain (PG) with 59 amino acids, a catalytic domain (CA) with 257 amino acids, a signal peptide domain (37 amino acids) that is cleaved during enzyme maturation, a transmembrane domain (TM) with 20 amino acids, and a C-terminal intracellular domain with 25 amino acids (Figure 1). The catalytic domain of CA IX is a critical part of its function in regulating pH balance, especially under hypoxic conditions in tumors. This domain has Zn^2+^ ion at its active site, which is essential for the catalytic activity of the enzyme. Three histidine residues (His 94, 96, and 116) coordinate the zinc ion at the base of the active site cleft. Two distinct regions made of hydrophobic or hydrophilic amino acids delimit the active site. In particular, Leu91, Val121, Val131, Leu135, Leu141, Val143, Leu198, and Pro202 define the hydrophobic region, while Asn62, His64, Ser65, Gln67, Thr69, and Gln92 identify the hydrophilic one [18,19].

Currently three different types of therapeutics have been developed: small-molecule inhibitors, monoclonal antibodies, and nanoparticle-based inhibitors [20,21,22,23,24]. Among these three small molecular inhibitors, sulfonamides are the most extensively studied, making them a promising class of inhibitors [25]. Antitumor activity of sulfonamides has been demonstrated in cells from various human tumors. These drugs inhibit hCA IX by coordinating with zinc ion, disrupting enzyme function and preventing CO_2_ conversion to bicarbonate, crucial for tumor growth and metastasis [26,27,28]. Sulfonamide drugs effectively inhibit tumor-associated hCA IX, but they lack selective targeting over thehuman carbpnic anhydrase II (hCA II) isoform in normal tissues, raising concerns about potential systemic toxicity [29,30,31]. For example, the SLC-0111 sulfonamide inhibitor has shown promise in the treatment of metastatic pancreatic cancer, a highly aggressive and difficult-to-treat cancer. SLC-0111 is in Phase 1b/II clinical trials in combination with gemcitabine, a chemotherapy drug for pancreatic cancer. The combination therapy aims to enhance gemcitabine therapeutic effects and control tumor growth. Preclinical studies showed improved anticancer efficacy due to SLC-0111′s high specificity for hCA IX, reducing off-target effects [32,33,34]. Despite these challenges, sulfonamides’ potent anticancer properties make them a focus of research, with efforts to improve selectivity and reduce toxicity, potentially expanding their therapeutic use in cancer treatment. Combining sulfonamides with other treatments or developing selective derivatives may reduce side effects while maintaining anticancer activity. Despite these advances, many known CA IX inhibitors, including SLC-0111, have limitations in terms of selectivity, potency under the tumor microenvironment, and potential systemic toxicity. Higher selectivity is especially crucial for reducing side effects while maintaining potent antitumor action.

In order to find selective inhibitors, we developed a 3D pharmacophore using two potential small molecule inhibitors 9FK (5-(1-naphthalen-1-yl-1,2,3-triazol-4-yl)thiophene-2-sulfonamide) and CJK (1-[(4-methylphenyl)methyl]-3-(2-oxidanyl-5-sulfamoyl-phenyl)urea). This approach aims to capture essential structural and functional features for the selective targeting of hCA IX, thereby guiding the design of new inhibitors with improved profiles. These potential inhibitors have a sulfonamide or sulfamate moiety able to coordinate the zinc ion of the catalytic binding site and inhibit enzymatic activity [26,27,28]. The constructed pharmacophore model identifies the key features necessary for binding to the active site of CA IX. We used a library of drug-like molecules and the DrugBank library to find promising inhibitors against CA IX. Compounds predicted by the pharmacophore model were then docked to evaluate for their binding affinity and interaction with the CA IX. To further assess the stability and binding strength of the complexes, molecular dynamics (MDs) simulations and MM-PBSA were applied, thereby identifying the most promising selective candidates.

## 2. Results

### 2.1. Pharmacophore Model-Based Screening

**Figure 2:**
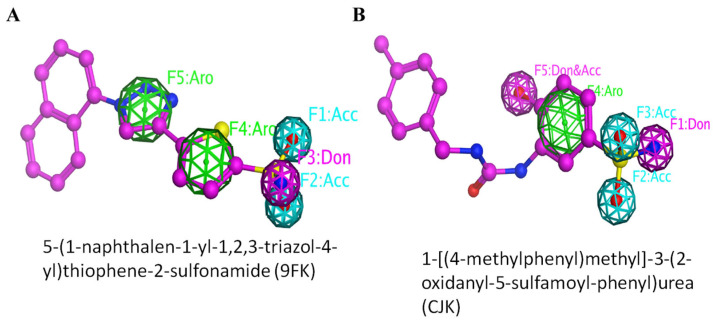
Pharmacophore models prepared based on the known inhibitors (**A**) 5-(1-naphthalen-1-yl-1,2,3-triazol-4-yl)thiophene-2-sulfonamide and (**B**) CJK. The features are represented as follows: Aro = aromatic ring (green), Acc = hydrogen bond acceptor (cyan), Don = hydrogen bond donor (magenta), Don&Acc = dual hydrogen bond donor/acceptor (pink mesh).

Two pharmacophore-based searches were performed against the ZINC library of drug-like molecules and the DrugBank library. The pharmacophore models include important features required for interactions with the CA IX active site. Pharmacophore model 1 was designed based on ligand 5-(1-naphthalen-1-yl-1,2,3-triazol-4-yl)thiophene-2-sulfonamide and pharmacophore model 2 was designed based on CJK. The pharmacophoric features were selected based on interactions with the buried residues of the CA IX active site, while features corresponding to parts of the ligand-facing solvent or were outside the pocket were eliminated, since they are unlikely to contribute to binding (Figure 2). Both ligands share a sulfonamide moiety which is buried deeply inside the active site. This buried nature of the sulfonamide tail in the ligands allows it to create strong hydrogen bonds and other interactions with the surrounding amino acid residues, which contribute greatly to binding affinity and specificity. The features included in the pharmacophore models were carefully chosen to highlight the functional significance of interacting regions of the pocket. This selective strategy improved the screening process by choosing compounds that are more likely to bind efficiently to the target. Pharmacophore model 1 screening generated six hits, eight against the DrugBank and ZINC library, respectively. On the other hand, the second pharmacophore model generated 14 hits against the DrugBank library and 552 against the ZINC library. These pharmacophore model searches generated a total of 580 compounds.

### 2.2. Molecular Docking

**Figure 3:**
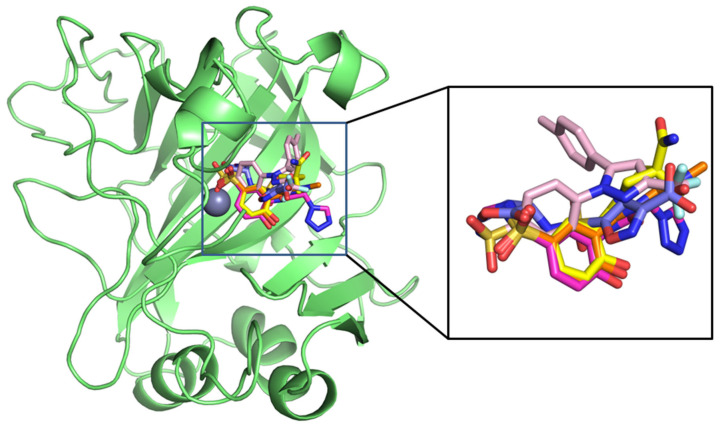
Docked conformations of the candidate ligands inside the active site of CA IX.

**Figure 4:**
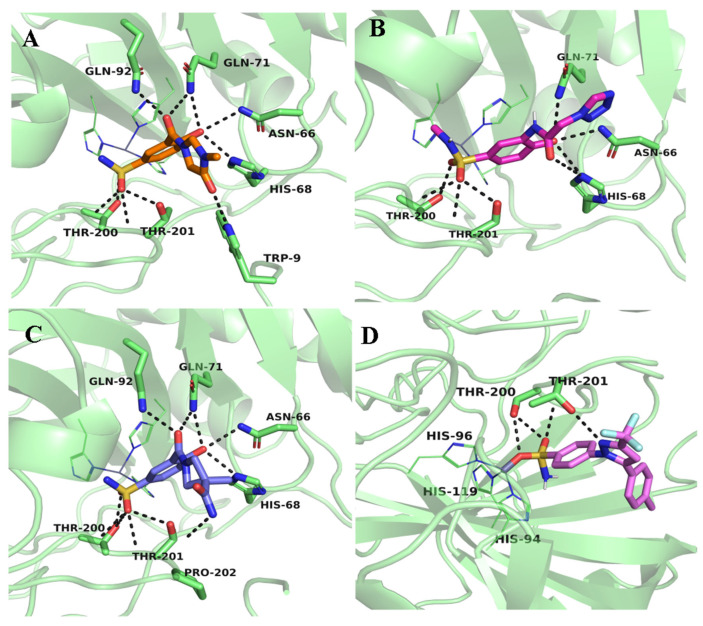
D representation of hydrogen binding interactions between CA IX and selected ligands. Hydrogen bonds are shown as black dotted lines. (**A**) ZINC613262012, (**B**) ZINC427910039, (**C**) ZINC616453231, and (**D**) DB00482.

After pharmacophore model-based screening, the screened compounds were docked into the active site of CA IX using AutoDock Vina. For docking experiments, we used the crystallized structure of CA IX (PDB ID 5FL4) (https://www.rcsb.org/structure/5fl4 (accessed on 13 October 2024)) complexed with 9FK (5-(1-naphthalen-1-yl-1,2,3-triazol-4-yl)thiophene-2-sulfonamide). During docking experiments, the protein was kept rigid, and only the ligand was allowed to be flexible. AutoDock Vina handles ligands in a flexible manner, enabling their rotatable bonds to sample numerous conformations throughout the docking process. This is accomplished by an iterative process in which the algorithm tests various ligand poses to determine the best binding orientation in the receptor’s binding region. In order to conduct a comparative analysis, potential known inhibitors of CA IX 9FK and CJK were also docked to the CA IX. Both compounds form hydrogen bond interactions with the residue Thr200 and Thr201, with binding energy −8.2 and −8.3 kcal/mol, respectively. The sulfonamide tail of the compounds forms interactions with the active site Zn2 ion, thereby blocking the enzyme’s catalytic function (Figure S1). After docking, the top 20 compounds were visualized and inspected. Four compounds ZINC613262012, ZINC427910039, ZINC616453231, and DB00482, having interactions with the crucial active site residues Thr200 and Thr201, were selected. All four candidate ligands occupied the same binding position inside the active site as the reference drugs (Figure 3). The docking results illustrated in Figure 3 and Figure 4 and Table 1 demonstrate the binding interactions between the selected ligands and the CA IX. All the compounds formed several hydrogen bonds, particularly with the residues Thr200 and Thr203, which are essential for stabilizing the ligand in the binding pocket. The proximity of the ligand to these residues suggests a strong interaction, enhancing its potential as an inhibitor. The presence of multiple hydrogen bonds between the ligand and the protein backbone enhances the binding affinity, contributing to the ligand’s effectiveness as a competitive inhibitor.

**Figure 5:**
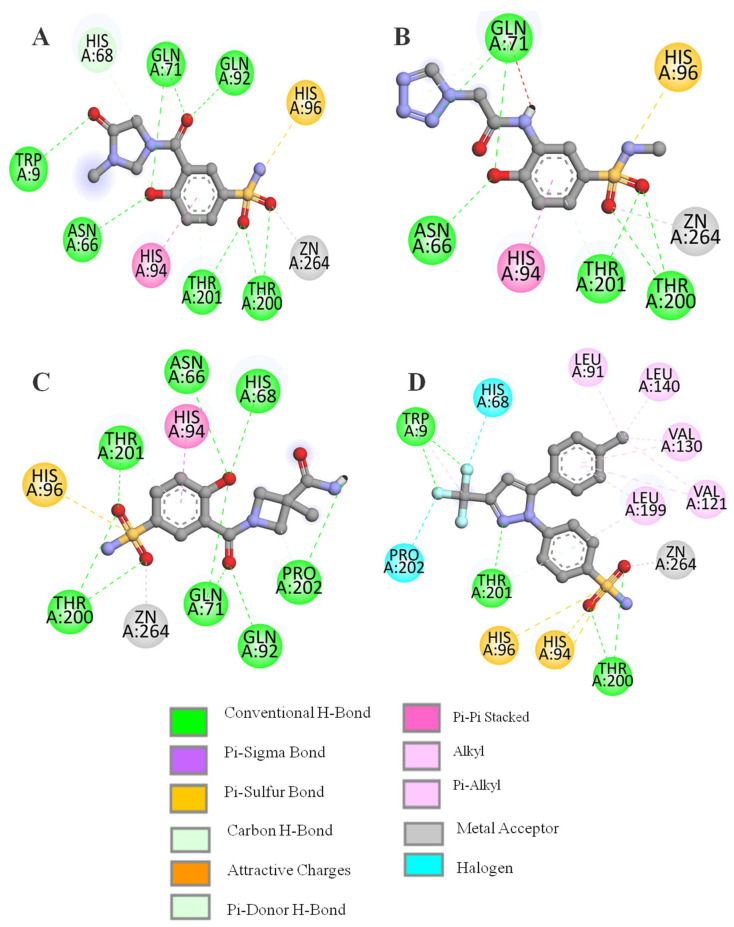
D interactions of the candidate ligands with target protein CA IX. (**A**) ZINC613262012, (**B**) ZINC427910039, (**C**) ZINC616453231, and (**D**) DB00482.

The sulfonamide group of the ligand ZINC613262012 demonstrated a significant binding affinity by forming four hydrogen bonds with the side chains of Thr200 and Thr201, with a docking score of −8.0 kcal/mol. Additionally, the hydroxyl group attached to the benzene ring established hydrogen bonds with the polar residues Asn66, His68, and Gln71, further stabilizing the binding of the ligand to the target protein. The OH group of the imidazole ring formed a hydrogen bond with Trp9. Similarly, the sulfonamide group of ZINC427910039 also formed hydrogen bond interactions with residues Thr200 and Thr201. Similarly, the hydroxyl group of the benzene ring created multiple hydrogen bonds with Asn66, His68, and Gln71, mimicking the interactions observed with other ligands. The sulfonamide group of the ligand ZINC616453231 also made hydrogen bonds with Thr200 and Thr201, with a docking score of −8.1 kcal/mol. Additionally the OH groups of the benzene ring formed hydrogen bonds with Asn66, His68, Gln71, and Gln92. Apart from hydrogen bonding, all these ligands also participated in hydrophobic interactions, which played a pivotal role in stabilizing the ligand–protein complex. The ligand DB00482 exhibited strong interactions with the target protein, primarily forming hydrogen bonds with Thr200, and Thr201. Specifically, the sulfonamide moiety engaged in strong interactions with the Zn^2+^ ion inside the active site and Thr 200 with a docking score of −8.7 kcal/mol. The Nitrogen from the pyrazole ring formed a hydrogen bond with the Thr201, while the methylphenyle ring formed various hydrophobic interactions with Val121, Val130, Leu 91, Leu140, and Leu199 (Figure 4 and Figure 5).

### 2.3. Physiochemical Characteristics

For a comparison study, essential physicochemical properties such as molecular weight (MW), topological polar surface area (TPSA), hydrogen bond donors (HBD), and hydrogen bond acceptors (HBA) were computed for all candidate compounds, as well as the reference drugs 9FK and CJK (Table 2). These factors represent key molecular characteristics and overall structural similarity between molecules.

**Figure 6:**
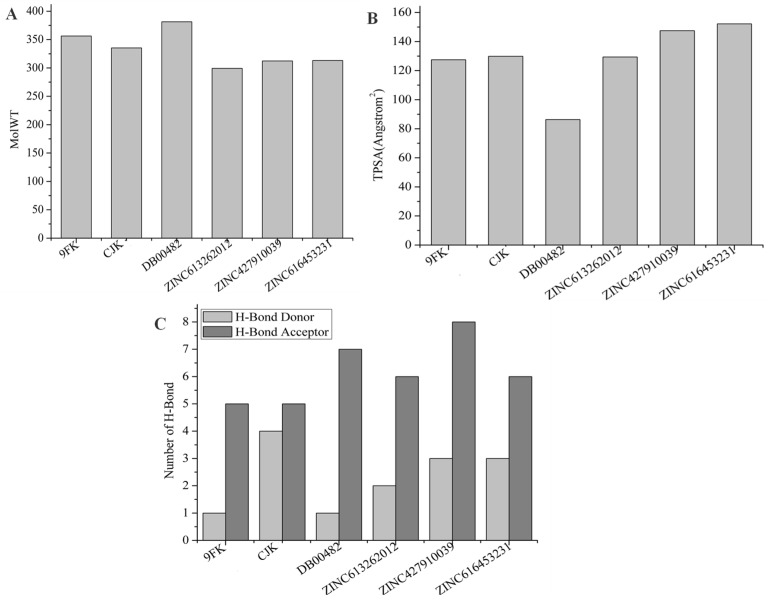
Physiochemical properties of the reference drugs and candidate ligands. (**A**) Molecular weight, (**B**) topological surface area (TPSA), and (**C**) number of hydrogen bond donors and acceptors.

**Figure 7:**
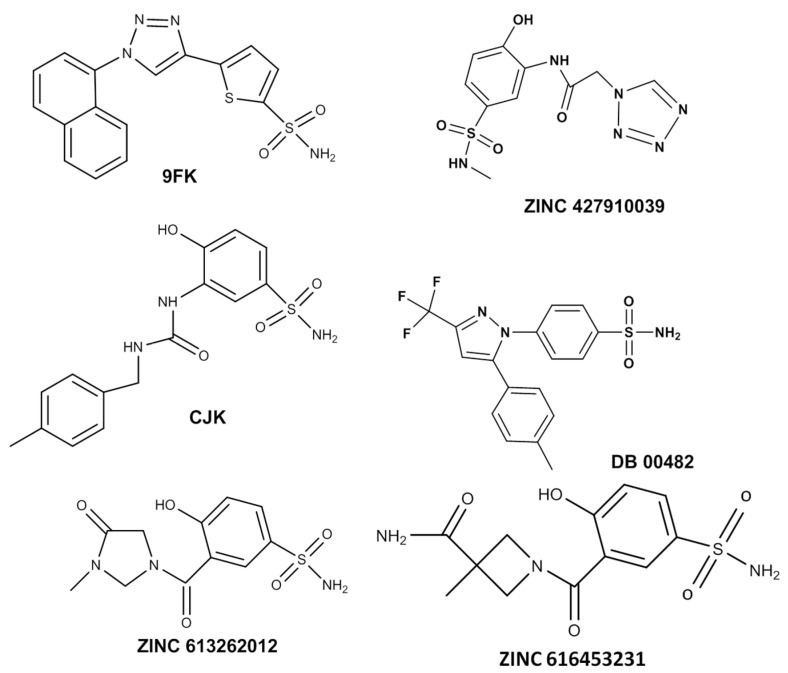
Chemical structure of the candidate ligands and the reference drugs 9FK and CJK.

The molecular weight and TPSA values of the candidate compounds remain relatively close to those of the reference drugs, indicating that these properties are not highly variable. This suggests that the candidate compounds retain a favorable balance between size and surface area, which could support good membrane permeability and bioavailability (Figure 6). Additionally, the hydrogen bond donor and acceptor counts of the candidate compounds are comparable to CJK and 9FK, implying a similar interaction potential with the target sites. Furthermore, the chemical structure of the candidate ligands (Figure 7) features a sulfonamide tail, analogous to that present in the reference drugs, which may contribute to the overall interaction profile and potential efficacy of these compounds.

### 2.4. Toxicity Profile

The toxicity profiles of the candidate compounds were evaluated. For Hepatotoxicity, all the candidate compounds, as well as the reference drugs, were classified as inactive, except for compound ZINC616453231, which was found to be active with a Hepatotoxicity score of 0.51. In terms of neurotoxicity, Immogenicity, cytotoxicity, Mutagenicity, and carcinogenicity, all candidate compounds showed inactive results, similar to the reference drugs, with the exception of the reference drug CJK, which was classified as active for carcinogenicity. The candidate compounds mostly fell within toxicity Class 4 and Class 5, similar to the reference drugs. Compounds DB00482, ZINC613262012, and ZINC616453231 fell within toxicity Class 4, while compound ZINC427910039 fell within Class 5. The LD50 values for the candidate compounds ranged from 1000 mg/kg to 5000 mg/kg. Compound ZINC613262012 had the highest LD50 of 5000 mg/kg, indicating a relatively higher safety margin when compared to other compounds. Overall, the candidate compounds demonstrated favorable toxicity profiles (Table 3).

### 2.5. Molecular Dynamics Simulations

Molecular dynamics (MD) simulations have become crucial in current drug development, providing novel insights into protein dynamics and function. These simulations allow researchers to investigate how proteins interact with small molecules, resulting in a better understanding of binding mechanisms and biological processes. By bridging the gaps left by experimental techniques, MD simulations enable the investigation of chemical and biological systems at resolutions that would otherwise be difficult to obtain. With this unprecedented potential, MD simulations are a useful tool for furthering the design and development of novel treatments [35,36].

**Figure 8:**
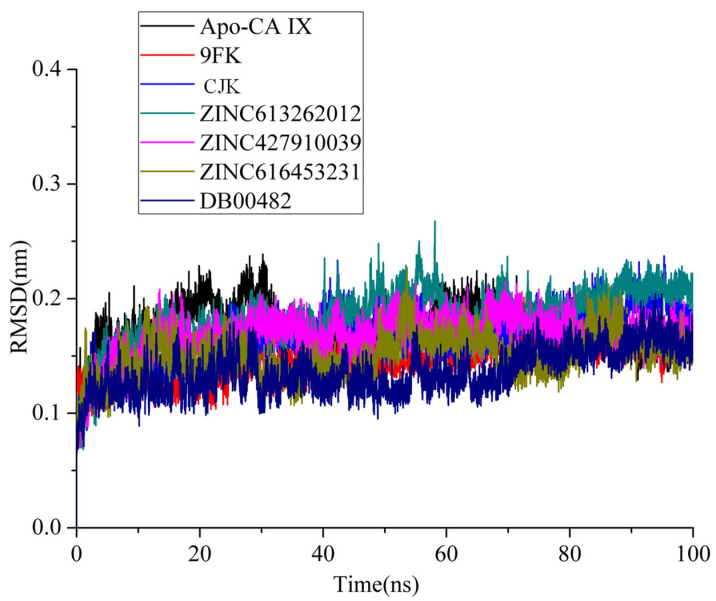
RMSD analysis of all the simulated systems over the time of 100 ns.

To assess the stability of the complexes, conformational adjustments, and structure dynamics, molecular dynamics simulations of the selected compounds and reported inhibitors (9FK and CJK) of CA IX were performed. A root mean square deviation analysis of all the simulated systems was plotted for all the frames across 100 ns time (Figure 8). The Apo form of CA IX had an average RMSD of 0.17 nm, whereas the known inhibitors 9FK and CJK had average RMSD values of 0.15 and 0.16 nm, respectively. The selected four compounds exhibited a good stability, with an average RMSD of 0.18 nm (ZINC613262012), 0.15 nm (ZINC427910039), 0.15 nm (ZINC616453231), and 0.13 nm (DB00482). All the simulated complexes exhibited RMSD values less than 0.25 nm. It can be seen from Figure 8 that all the complexes remained stable throughout the simulation. Slight fluctuations in the RMSD pattern of ZINC616453231 were observed, but the RMSD values remained below 0.25 nm. A slight increase in the RMSD values of the complexes 9FK, CJK, ZINC613262012, ZINC427910039, and DB00482 were observed at 60 ns, but after that, all the systems maintained a steady state and remained stable until the end of the simulations. The comparative analysis revealed that no major fluctuations were observed in all of the systems. All the systems maintained stable conformations.

**Figure 9:**
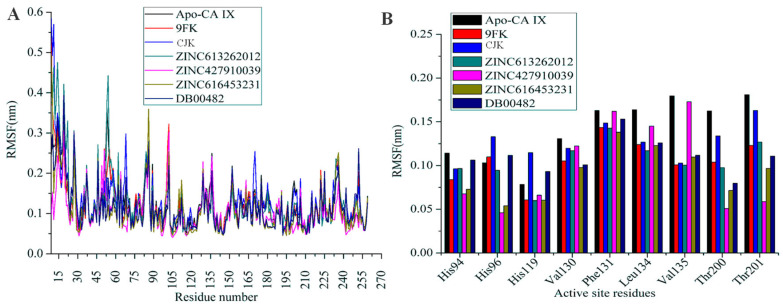
RMSF analysis of the simulated systems. (**A**) RMSF of all the systems plotted as residue numbers on the X-axis and RMSF on the Y-axis. The residue number sequence starts from 9, as in the PDB ID 5FL4. (**B**) RMSF values of the active site residues of all the systems.

Root mean square fluctuations (RMSF) calculate the average positional variation in every residue in a molecule during the course of the simulation. It sheds light on the stability and dynamics of different regions of a protein. Less RMSF values indicate stability while high RMSF values indicate larger fluctuations. Figure 9A illustrates the RMSF for the Apo-CA IX structure, known inhibitors, and the selected compounds. It can be observed that the Apo-CA IX structure shows higher fluctuations in certain regions compared to the ligand-bound systems, suggesting that ligand binding stabilizes the protein. The top screened compounds demonstrate RMSF profiles comparable to those of the known inhibitors, indicating similar levels of stability and effective interactions with the protein. There were few residues exhibiting fluctuations larger than 0.25 nm, which corresponds to the loops of the CA IX structure. The Apo form of CA IX, 9FK, CJK, and ZINC613262012 had an average RMSF of 0.12 nm. Whereas compounds ZINC427910039 and ZINC616453231 had an average RMSF of 0.10 nm (Table 4), and DB00482 had an average RMSF of 0.11 nm. Figure 9B focuses on the RMSF of active site residues. It reveals that the most active site residues exhibit low flexibility in the complexes compared to the Apo form. Among the ligands, ZINC616453231 and ZINC613262012 show minimal fluctuation at key active site residues, suggesting strong and stable interactions with the CA IX active site. The known inhibitors 9FK and CJK also display similar stabilization effects, further validating the performance of the selected compounds.

### 2.6. Intermolecular Interactions

**Figure 10:**
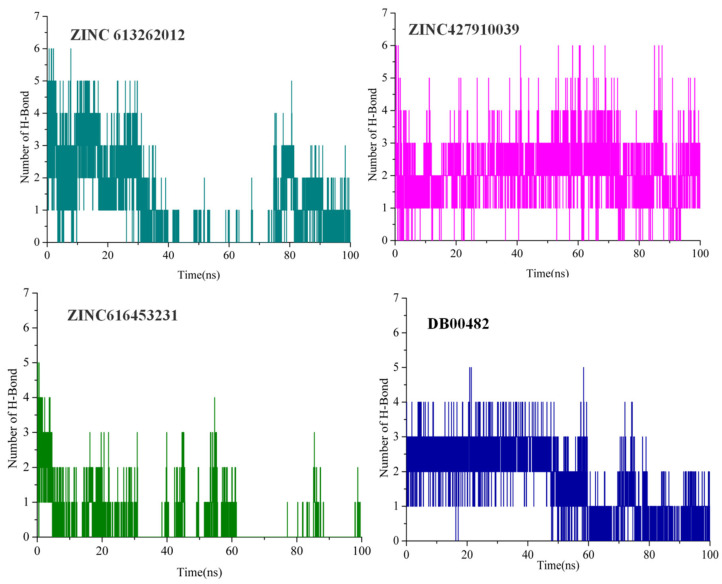
Hydrogen bonds formed between ligands and CA IX proteins over the 100 ns simulation time.

To confirm the stability of the complexes, the number of hydrogen bonds paired within 0.35 nm between CA IX and the docked compound were estimated during MD simulations. Hydrogen bond formation patterns are shown in Figure 10 for the four selected compounds. The pattern of hydrogen bond formation revealed diverse patterns of contact stability. ZINC427910039 and DB00482 show more constant hydrogen bonding throughout the simulation, indicating persistent interactions over time. For ZINC613262012, the number of hydrogen bonds varies greatly between 0 and 6, with a greater frequency of bond creation reported within the first 30 nanoseconds. This trend may indicate an initial strong binding mode that becomes less stable as the simulation progresses, possibly due to conformational rearrangements of either the ligand or protein. ZINC616453231 exhibited the lowest and most inconsistent hydrogen bond profile among the four complexes. The number of hydrogen bonds was usually below two, only reaching four bonds in the first 40 ns during some time frames. After that, the bonding dropped sharply, often showing just one or no bonds until the end of the simulation. Overall, ZINC613262012, DB00482, and ZINC427910039 exhibited a more stable hydrogen bonding pattern, maintaining higher numbers of hydrogen bonds throughout the simulation compared to the other compounds.

### 2.7. MM-PBSA Analysis

The binding free energy calculations were performed for known inhibitors and the candidate compounds using the MM-PBSA approach to evaluate their binding affinities to the target protein. The binding free energy components are presented in Table 5 including the contributions of different components. Among all, CJK and ZINC616453231 demonstrated weaker binding affinities with ΔTOTAL −5.62 kcal/mol and −1.28 kcal/mol, respectively, suggesting that these compounds lack strong stabilizing interactions, particularly in the van der Waals and electrostatic components. These binding free energy results of compound ZINC616453231 are consistent with the above MD simulation analysis. Meanwhile, ZINC427910039 and DB00482 showed the most favorable binding affinity with ΔTOTAL −18.77 kcal/mol −12.29 kcal/mol, respectively. This high binding affinity of ZINC427910039 was due to its significant van der Waals ΔEvdW (−28.81 kcal/mol) and electrostatic ΔEelec (−26.60 kcal/mol) contributions, as well as the strong stabilization from solvation energy ΔGSOLV (36.64 kcal/mol), whereas complex ZINC613262012 exhibited a binding affinity similar to the known inhibitor 9FK (ΔTOTAL −10.92 kcal/mol). The binding of this compound had balanced contributions from gas-phase and solvation energies, with noticeable van der Waals interactions. Notably, the binding free energies of these selected inhibitors (ZINC427910039, ZINC613262012, and DB00482) surpassed that of the 9FK known inhibitor. This observation highlights the potential of the selected inhibitors as promising candidates for further experimental evaluation and optimization in the context of therapeutic development.

### 2.8. DFT Analysis of the Three Selected Candidate Compounds

**Figure 11:**
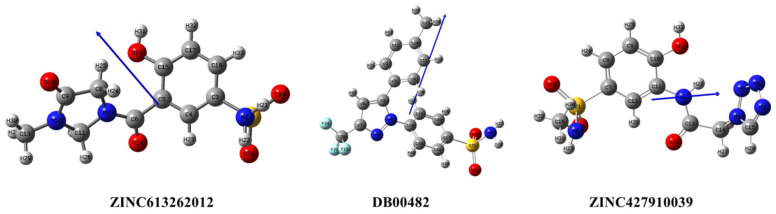
Optimized structure of lead compounds.

The density functional theory computations gave in-depth information on structural, electronic, and reactivity properties of the studied compounds. The optimized geometries, frontier orbital analysis, and molecular electrostatic potential mapping made it possible to examine the physicochemical behavior of ZINC613262012, DB00482, and ZINC427910039 in detail. The parameters are also critical to interpret molecular stability as well as the implications of the interactions of the compounds with biological macromolecules. The compounds’ optimized structures are depicted in Figure 11. Molecules all reached stable geometries with no imaginary frequencies, indicating that the molecules lie in true local minima on the potential energy surface. The optimized conformations showed a favorable distribution of electronic density around the molecular skeleton that has helped in their overall thermodynamic stability. These optimized geometries form the backbone for the following electronic property calculations, because slight conformational changes can have a major effect on the HOMO LUMO distributions of the electrostatic potential surfaces [37].

#### 2.8.1. FMO Analysis

**Figure 12:**
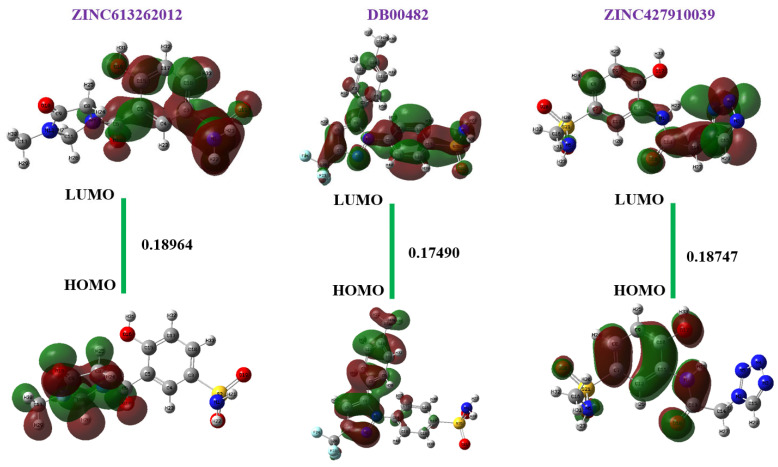
HOMO, LUMO, and energy gap of lead compounds.

Table 6 listed all the HOMO and LUMO energies and the estimated energy gaps, as well as Figure 12, which shows the same. The HOMO was between −0.26193 and −0.25681 eV and the LUMO varied between −0.08551 to −0.06934 eV. The HOMO-LUMO gaps suggested all the compounds are chemically reactive and have an enhanced charge transfer capability (0.17490–0.18964 eV). DB00482 had the least gapping (0.17490 eV) amongst the molecules, indicating further suitability for electron excitation and interaction with biological targets. In all three compounds, the comparatively close energy level between the HOMO and LUMO suggests that the compounds may easily undergo electronic transitions, and this is desirable in drug–target interactions [38].

#### 2.8.2. Dipole Moment and Electronic Energy

Values of the dipole moment showed marked variations in value which indicated that there were differences in the polarity of the molecules. The greatest dipole moment was seen in B00482 (8.79 Debye), which in turn would indicate an increased solubility in polar media and potential to act as stronger hydrogen bonds or dipole–dipole interactions with biomolecules. ZINC613262012 and ZINC427910039 had slightly smaller dipole moments (5.00 Debye and 3.97 Debye, respectively) suggesting that they behaved more modestly polar. The calculated total electronic energies (=−1365.33–1668.44 Hartree) indicated that all the molecules are in thermodynamically stable positions, giving confidence in the accuracy of the structures obtained [39].

#### 2.8.3. Molecular Electrostatic Potential (MEP)

**Figure 13:**
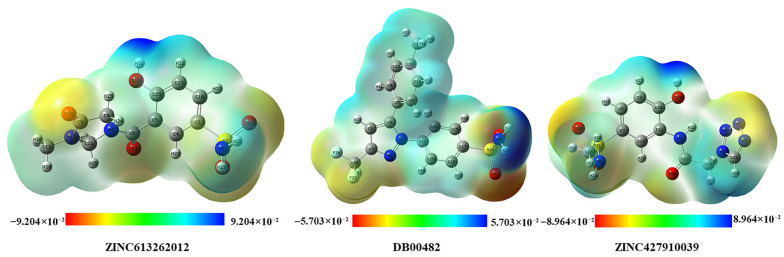
MEP structure and scale of lead compounds.

At the B3LYP/6-311G level, molecular electrostatic potential (MEP) maps were generated to illustrate the attractiveness of ZINC613262012, DB00482, and ZINC427910039 surfaces. Figure 13 shows that the MEP plots give clear-cut results in terms of identifying light-seeking, electron-deficient areas (blue) and light-avoiding, electron-rich portions (red) of the molecules. The red areas show where electrophilic attack is likely to be, and the blue areas the probable locations of nucleophilic attack [40]. The three compounds exhibited distinct areas of the positive and negative electrostatic potential that supported the idea of the existence of chemically active regions that can be involved in the non-covalent interaction with biological macromolecules. The studied compound with the most pronounced polarity was DB00482, which also had the highest dipole moment, implying its ability to readily form hydrogen bonds and electrostatic interactions with proteins. The MEP distribution similarly augments the HOMO-LUMO and reactivity descriptors in terms of identifying localized reactive sites that may become central interactions sites during ligand target recognition. Therefore, the MEPs surfaces allow them to learn important details regarding the possible binding mode of the substances, and this fact testifies to their value as potential candidates for more biological investigations [41].

All compounds were thermodynamically stable, as affirmed by geometry optimization and with an FMO analysis showing small HOMO-LUMO excitation energies, which imply a high chemical reactivity and possible charge transfer. The studied molecules that were ranked as DB00482 had the lowest energy gap, the largest dipole moment, and the highest electrophilic character, which indicated that this molecule would be more likely to have a polar interaction with the biomolecular targets. Conversely, ZINC427910039 showed a more nucleophilic profile, providing a complementary reactivity that could preferably interact with biological systems as well. The global reactivity descriptors that include the molecular electrostatic potential (MEP) mapping helped identify clear reactive sites, which may serve as a non-covalent bonding site [42]. In general, these results prove that the studied substances have potential electronic and reaction patterns.

### 2.9. Extended Simulation of the ZINC427910039 and DB00482

**Figure 14:**
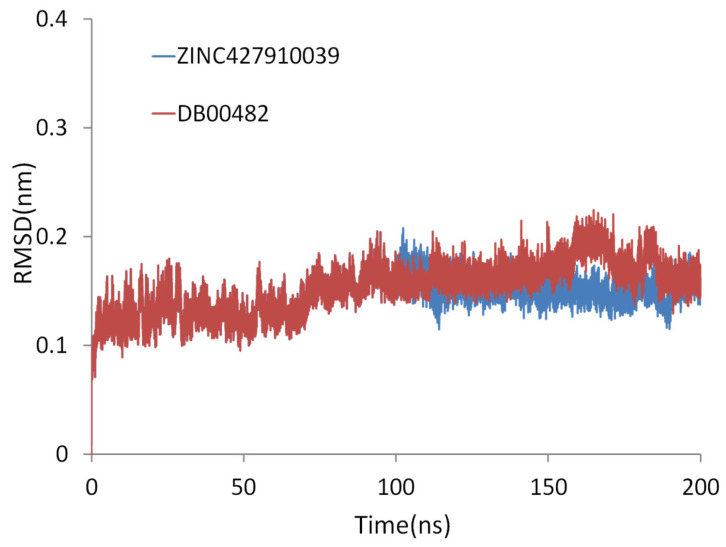
Extended 200 ns molecular dynamics simulation results of ZINC427910039 and DB00482 with CA IX.

To further validate the stability of the ligand–protein complexes beyond the initial 100 ns simulation, extended molecular dynamics (MD) simulations of 200 ns were performed for the two selected compounds, ZINC427910039 and DB00482, in complex with CA IX (Figure 14). The extended trajectories confirmed the robustness of the binding interactions observed in the shorter runs. For ZINC427910039, the RMSD profile remained stable below 0.2 nm throughout the 200 ns simulation, suggesting that the complex retained conformational stability. For DB00482, the RMSD plot remained stable throughout the 200 ns simulation, with fluctuations consistently below 0.2 nm, indicating a highly stable complex with CA IX. The ligand preserved its binding orientation within the active site, maintaining key interactions during the entire trajectory. Overall, the extended simulations reinforce the conclusion that both ZINC427910039 and DB00482 remained stable in complexes with the ligands, thereby supporting their potential as selective CA IX inhibitors.

### 2.10. Comparative Docking Analysis of CA IX and Off-Target Isoforms (CA I, CA II)

To further validate the top three ligands identified through MM-GBSA analysis, we performed comparative docking with CA I and CA II. Docking experiments revealed that all three potential ligands showed better binding to CA IX than to the off-target isoforms CA I and CA II. In CA IX, ZINC613262012 and ZINC427910039 both had docking scores of −8.0 kcal/mol, while DB00482 showed the strongest interaction with a score of −8.7 kcal/mol (Table 1). When compared with CA II, the same compounds displayed slightly less favorable scores (−8.1, −7.8, and −8.4 kcal/mol, respectively), indicating a preference for CA IX binding. Against CA I, the ligands exhibited lower affinities (−7.2, −7.3, and −7.6 kcal/mol), again confirming the stronger interactions with CA IX. These findings show that ZINC613262012, ZINC427910039, and DB00482 are potential inhibitors with a strong binding strength and significant selectivity for CA IX over CA I and CA II (Table 7).

## 3. Discussion

Carbonic anhydrase IX (CA IX) has substantial therapeutic promise in cancer therapy due to its involvement in tumor hypoxia. CA IX, a hypoxia-induced protein, maintains pH homeostasis in the tumor microenvironment, boosting tumor cell survival and invasiveness under acidic environments. Its selective expression on hypoxic tumor cells and low presence in normal tissues make it a promising target. Different targeted therapeutic approaches such as small-molecule inhibitors and immunotherapy have been practiced against this target [25,43,44].Various small-molecule inhibitors and sulfonamides have been discovered against CA IX [25]. These inhibitors inhibit CA IX by coordinating with zinc ion, disrupting enzyme function, and preventing the CO_2_ conversion to bicarbonate, which is crucial for tumor growth and metastasis [26,27,28]. The SLC-0111 sulfonamide inhibitor has shown promise in the treatment of metastatic pancreatic cancer. SLC-0111 is in Phase 1b/II clinical trials in combination with gemcitabine, a chemotherapy drug for pancreatic cancer [32,33,34]. Despite these challenges, sulfonamides’ potent anticancer properties make them a focus of research, with efforts to improve selectivity and reduce toxicity, potentially expanding their therapeutic use in cancer treatment.

In silico drug discovery methods such as molecular modeling and molecular dynamics (MDs) simulations have become indispensable in current drug discovery processes. These computational approaches are widely used in all stages of drug development, from lead identification to optimization and drug–target interactions. The primary benefit is their capacity to dramatically cut the time and expenses associated with traditional drug development pipelines [45,46]. This study was aimed at identifying potential inhibitors targeting CA IX. For this purpose, a pharmacophore model based on the two well-known inhibitors of CA IX was built, mimicking the essential features required to interact with the active site of the CA IX. The created pharmacophore model was then used to perform virtual screening against the ZINC library of drug-like compounds and the DrugBank database. To evaluate binding conformations and interactions, selected hits were docked into CA IX’s active site. Molecular docking analysis identified four candidate inhibitors that interact with crucial active site residues occupying the same binding position as the native inhibitor. For comparison, the study included two well-known inhibitors, CJK and 9FK. Both molecules, which include a sulfonamide tail, make hydrogen bonds with residues Thr200 and Thr201. Interestingly, the candidate compounds also interacted with these residues while simultaneously forming hydrogen bonds with other important residues in the active site [47]. Furthermore, the in silico validation of these putative inhibitors was performed through (MD) simulations and MM-PBSA. The root mean square deviation (RMSD) and root mean square fluctuation (RMSF) analysis revealed that all complexes were stable, with the exception of compound ZINC616453231, which showed minor fluctuations. The hydrogen bond pattern in Figure 10 indicated that compounds ZINC427910039, DB00482, and ZINC613262012 generated a greater number of stable hydrogen bonds, demonstrating the stability of these complexes. Compound DB00482 (Celecoxib), a selective COX-2 inhibitor, is primarily an NSAID with a reduced risk of gastrointestinal bleeding, used to manage arthritis pain symptoms and reduce precancerous polyps in the colon in familial adenomatous polyposis [48,49]. Additionally, this compound has also been repurposed for different types of malignancies. This drug fights cancer by inducing apoptosis, inhibiting angiogenesis, and modulating the tumor microenvironment. Furthermore, MM-PBSA simulations showed ZINC427910039, DB00482, and ZINC613262012 as potential CA IX inhibitors, with binding energies of −18.77 kcal/mol, −12.29 kcal/mol, and −10.92 kcal/mol, respectively. Notably, the binding free energy scores of the reported ligands were superior to those of the known inhibitors, highlighting their potential as effective therapeutic agents for CA IX inhibition. Moreover, comparative docking against CA I and CA II confirmed that these ligands preferentially bind to CA IX, underscoring their selectivity and therapeutic relevance. In addition to docking and MDs simulations, DFT-based analysis revealed important information about the electrical and reactivity profiles of the three selected compounds. The computed frontier molecular orbitals (HOMO-LUMO) showed small energy gaps, indicating that all compounds are chemically reactive and have a high charge transfer potential. Furthermore, global quantum reactivity descriptors such as chemical hardness, softness, electrophilicity, and nucleophilicity indicated that the molecules had a favorable balance of electronic properties, which contributed to their stability and ability to interact effectively with biological targets. These DFT results are consistent with the docking and MD results, indicating that the discovered compounds are electrically well-suited to act as possible CA IX inhibitors.

In silico techniques are valuable in rapidly screening compounds and providing detailed insights into their binding modes and interaction stability. These methods offer a cost-effective and time-efficient way to prioritize promising candidates for further investigation. However, the findings do not account for the full complexity of biological systems, including metabolism, off-target interactions, and physiological variability. Therefore, experimental validation is essential to confirm the inhibitory potential and therapeutic relevance of the identified compounds.

## 4. Methods

### 4.1. Pharmacophore Model Building

A pharmacophore model was developed based on the known inhibitors, 5-(1-naphthalen-1-yl-1,2,3-triazol-4-yl)thiophene-2-sulfonamide [47] and CJK (1-[(4-methylphenyl)methyl]-3-(2-oxidanyl-5-sulfamoyl-phenyl)urea) [50] targeting carbonic anhydrase IX (CA IX). The 3D structure of the ligand was obtained from the PubChem database and subjected to energy minimization using MMFF94 force fields to achieve a stable conformation. The optimized structure was then imported into MOE software 2022, where key features such as hydrogen bond donors, acceptors, aromatic rings, and hydrophobic regions were identified. These features were used to construct the initial pharmacophore model. Two pharmacophore models were constructed, one for 5-(1-naphthalen-1-yl-1,2,3-triazol-4-yl)thiophene-2-sulfonamide and one for CJK. The pharmacophoric features were selected based on interactions with the buried residues of the CA IX active site, while features corresponding to parts of the ligand protruding outside the binding pocket were not included. For the ligand 5-(1-naphthalen-1-yl-1,2,3-triazol-4-yl)thiophene-2-sulfonamide, five features were identified: F1 (acceptor), F2 (acceptor), F3 (donor), F4 (aromatic), and F5 (aromatic). Similarly, five features were identified for CJK. Of these, four features matched those of the first ligand. However, the fifth feature differed: it represented a combined donor and acceptor characteristic unique to the SLC-0111 binding profile. This differentiation highlights the subtle but significant variations in how each ligand interacts with the CA IX active site. These two models were then used to screen a library of 3 million drugs, like molecules from the ZINC database (https://zinc15.docking.org/ (accessed on 13 October 2024)) and 2800 FDA approved drugs from DrugBank (https://go.drugbank.com/ (accessed on 13 October 2024)).

### 4.2. Protein and Ligand Structure Optimization

After pharmacophore model-based screening, the screened ligands were optimized using the AutoDock Tools [51]. First, all the ligands were energy minimized using 500 steps of the steepest descent method. For this purpose, the MMFF94 force field implanted in Openbabel [52] was utilized. After that, all the ligands were processed by adding Gasteiger charges and polar hydrogens. After optimization, the ligands were saved in pdbqt format for docking purposes. The protein structure of carbonic anhydrase IX (CA IX) used in this study was retrieved from the protein data bank (PDB) under the accession ID 5FL4 with resolution 1.82A (https://www.rcsb.org/structure/5FL4 (accessed on 13 October 2024)). The structure was crystallized with the inhibitor 5-(1-naphthalen-1-yl-1,2,3-triazol-4-yl)thiophene-2-sulfonamide. All the molecules crystallized with CA IX were deleted, only the CA IX structure was kept. The CA IX target structure was optimized using AutoDock Tools-1.5.6 by adding charges and polar hydrogens.

### 4.3. Molecular Docking

The compounds screened using the pharmacophore models were then docked into the active site of CA IX with AutoDock Vina [53]. Ensuring an optimized search space, the docking procedure specified the dimensions of the grid box encompassing the binding site with a center of x = 11.928, y = −25.751, and z = 59.429. The docking box size was set to 35 Å for each x, y, and z axis to fully explore the binding search space. The exhaustiveness parameter was set to 16, which allows for a comprehensive search of the ligand conformations. For each ligand, twenty posed for each ligand were generated. Exhaustiveness was set to be 16. The exhaustiveness parameter controls the strength of the conformational search. Higher exhaustiveness values result in more extensive sampling of ligand conformations, which increases the chance of finding an optimum binding pose. Vina determines a binding affinity score for each conformation. After docking, the resultant docking conformations were then ordered based on binding scores and examined with PyMol (https://www.pymol.org/ (accessed on 13 October 2024)). This analysis includes an assessment of important interactions between the ligands and the protein, such as hydrogen bonds and hydrophobic contacts, which are necessary for understanding the binding affinity and specificity of the screened compounds.

### 4.4. Toxicity Measurement

The toxicity profiles of the compounds were evaluated using ProTox 3.0 (https://tox.charite.de/protox3/ (accessed on 13 October 2024)), a web-based tool for predicting the toxicological properties of small molecules. ProTox-II provides predicted toxic doses (LD50 values), toxicity classes, and potential risks for various toxicity types such as organ toxicity (Hepatotoxicity and neurotoxicity) and end-point toxicity (Immunotoxicity, carcinogenicity, Mutagenicity, and cytotoxicity).

### 4.5. Molecular Dynamics Simulations

Molecular dynamics simulations were performed in order to analyze the dynamic stability of the complexes. MD simulations were performed for the apo CA IX, candidate compounds, and the known inhibitors of CA IX (9FK and CJK). GROMACS 2022 [54] was used to conduct the MD simulations. The CHARMM force field was used for protein parameterization, and the CGenFF server (https://app.cgenff.com/ (accessed on 25 October 2024)) was used for ligand parameter generation. The systems were solvated in the dodecahedron box with a distance 1.0 nm from each side of the box edges. Subsequently, ions were added to neutralize the systems, and systems were energy minimized using the steepest descent method for 1000 steps. After energy minimization, the temperature of the systems was equilibrated using an NVT ensemble for 250 ps. Furthermore, the systems were simulated using the NPT ensemble for 250 ps. Finally, production of 100 ns was performed for each system band, and the trajectories were saved after every 2 fs.

### 4.6. Molecular Mechanics Poisson–Boltzmann Surface Area (MM-PBSA) Analysis

In order to validate the stability of the complexes analyzed through the MD simulations, the MM-PBSA method was employed. For this purpose, the gmx_MMPBSA module [55] was used to calculate the binding free energies of the complexes. Trajectories extracted from the last 25 ns consisting of 5000 frames were used to estimate the binding affinities. Gmx_MMPBSA estimates the binding free energy as follows:

$${∆G}_{binding}={∆E}_{MM}+{∆E}_{solv}-T∆S$$

$${∆E}_{MM}={∆E}_{bonded}+{∆E}_{elec}+{∆E}_{vdw}$$

$${∆E}_{solv}={∆E}_{polar}+{∆E}_{non-polar}$$

In the above equations

−T∆S denotes the entropic contribution.

${∆E}_{MM}$ includes the molecular mechanics potential energy comprising van der Waals (ΔE_vdw_) energy and electrostatic (ΔE_elec_).

${∆E}_{solv}\;$is the solvation-free energy

${∆E}_{polar}$ and ${∆E}_{non-polar}$ represent the polar and non-polar solvation energies, respectively.

### 4.7. Density Functional Theory (DFT)

Gaussian software-based DFT was employed to investigate the sensitivity of the electronic characteristics of the lead molecules to the target protein. Better convergence of the individual molecular structures has been achieved by optimizing the geometry using the B3LYP hybrid functional and 6- 311G basis set [56]. This was selected because it constitutes an excellent approach in predicting molecular proprieties and, at the same time, is not so expensive to employ. The program calculated all the results of optimized geometry, the HOMO LUMO and energy gap, and the dipole moment and the ESP maps in each molecule. The full set of less than or equal to first principles-based global reactivity descriptors, namely the ionization potential (I), electron affinity (A), chemical potential, electronegativity, chemical hardness, chemical softness (S), electrophilicity index (omega), nucleophilicity index (N), and the additional electronic charge transfer, were computed as per Koopmans’ theorem [57]. The data that they provide aids in the understanding of the reactions and impacts of the substances which are used in the procedure of molecular docking and binding.

## 5. Conclusions

The aim of this study was to identify potential inhibitors targeting carbonic anhydrase IXCA IX to knockdown its expression and inhibiting its activity. Through pharmacophore model-based screening and molecular docking, four promising compounds were identified that demonstrated effective key interactions with the key active site residues, comparable to known inhibitors (9FK and CJK). A detailed interaction analysis revealed that these compounds interacted effectively with key active site residues, similar to known inhibitors, and formed a higher number of stable hydrogen bonds. Molecular dynamics (MD) simulations further confirmed the stability of these complexes followed by MM-GBSA. MM-PBSA free energy calculations indicated favorable binding affinities, with values of −18.77 kcal/mol for ZINC427910039, −10.92 kcal/mol for ZINC613262012, and −12.29 kcal/mol for DB00482. Furthermore, DFT-based frontier molecular orbital and global reactivity analyses revealed that all three compounds had small HOMO-LUMO gaps, good chemical softness, and a favorable electrophilicity/nucleophilicity balance, indicating their chemical reactivity and stability. Overall, these electrical properties complement the docking and MD results, further supporting the potential of the selected compounds as promising CA IX inhibitors. Importantly, comparative docking confirmed that these ligands exhibited stronger binding towards CA IX over off-target isoforms carbonic anhydrase I (CA I) and carbonic anhydrase II (CA II). This observed selectivity further enhances their potential as specific CA IX inhibitors for future clinical evaluation. These findings suggest that these identified compounds can be considered for further clinical evaluation.

## Figures and Tables

**Table 1:** Docking scores and residues making hydrogen bonds are presented for the reference drugs and selected ligands.

Compound	Binding Score Kcal/mol	Residues Involved in H-Bonds	Total Number of H-Bonds Formed
9FK	−8.2	Thr200, Thr201	5
CJK	−8.3	Thr200	2
ZINC613262012	−8.0	Trp9, Gln71, Asn66, His86, Gln92, Thr200, and Thr201	10
ZINC427910039	−8.0	Gln71, Asn66, Thr200, and Thr201	8
ZINC616453231	−8.1	Asn66, His68, Gln71, Gln92, Thr200, Thr201, and pro202	11
DrugBank ID (DB00482)	−8.7	Thr200, Thr201	4

**Table 2:** Physiochemical properties of reference and candidate compounds.

Compound	Molecular Weight (g/mol)	LogP	H-Bond Donors	H-Bond Acceptors	TPSA (Å^2^)
9FK	356.432	2.796	1	6	127
CJK	335.385	1.67	4	5	130
ZINC613262012	299.308	−1.089	1	5	123
ZINC427910039	312.311	−1.075	3	8	139
ZINC616453231	313.335	−1.013	2	5	146
DB00482	381.379	3.514	1	4	77

**Table 3:** Toxicity profile of the reference drugs and selected ligand.

Toxicity	9FK	CJK	ZINC 613262012	ZINC 427910039	ZINC 616453231	DB00482
Hepatotoxicity	Inactive	Inactive	Inactive	Active (0.51)	Inactive	Inactive
Neurotoxicity	Inactive	Inactive	Inactive	Inactive	Inactive	Inactive
Immogenicity	Inactive	Inactive	Inactive	Inactive	Inactive	Inactive
Carcinogenicity	Active	Inactive	Inactive	Inactive	Inactive	Inactive
Cytotoxicity	Inactive	Inactive	Inactive	Inactive	Inactive	Inactive
Mutagenicity	Inactive	Inactive	Inactive	Inactive	Inactive	Inactive
Toxicity Class	4	5	4	5	4	4
LD50	400 mg/kg	5000 mg/kg	1000 mg/kg	3500 mg/kg	1000 mg/kg	1400 mg/kg

**Table 4:** This table presents the average RMSD values and average RMSF values of all the simulated systems.

Compound	RMSD (nm)	RMSF (nm)
Apo CA IX	0.174478	0.12
9FK	0.147564	0.12
CJK	0.166459	0.12
ZINC613262012	0.184293	0.12
ZINC427910039	0.158802	0.10
ZINC616453231	0.150055	0.10
DB00482	0.136467	0.11

**Table 5:** Binding free energy analysis of the known inhibitors and selected compounds. This table provides the energy contribution by different energy components in Kcal/mol.

Compounds	ΔEelec	ΔEvdw	ΔEGB	ΔESURF	ΔGSOLV	ΔGGAS	ΔTOTAL
9FK	−23.34	−12.29	26.84	−1.92	24.92	−35.63	−10.71
CJK	−3.70	−8.05	7.92	−1.16	6.13	−11.75	−5.62
ZINC613262012	−18.94	−15.36	25.83	−2.45	23.38	−34.30	−10.92
ZINC427910039	−26.60	−28.81	40.78	−4.14	36.64	−55.41	−18.77
ZINC616453231	−1.88	−2.85	3.81	−0.36	3.44	−4.72	−1.28
DB00482	−5.37	−23.01	19.92	−3.83	16.09	−28.38	−12.29

**Table 6:** HOMO, LUMO, energy gap, and the global quantum reactivities calculated at the DFT/B3LYP/6-311G level.

	Formula	ZINC613262012	DB00482	ZINC427910039
Dipole moment		5.009836	8.795973	3.979740
Electronic energy		−1365.331849	−1668.442782	−1415.560093
LUMO		−0.07229	−0.08551	−0.06934
HOMO		−0.26193	−0.26041	−0.25681
Energy gap (eV)	E_HOMO_ − E_LUMO_	0.18964	0.17490	0.18747
Electron affinity (A)	A = −E_LUMO_	0.07229	0.08551	0.06934
Ionization potential (I)	I = −E_HOMO_	0.26193	0.26041	0.25681
Chemical potential (μ)	μ = 1/2 (I + A)	0.16711	0.17296	0.16308
Electronegativity (χ)	χ = −1/2 (I + A)	−0.16711	−0.17296	−0.16308
Chemical hardness (η)	η = 1/2 (I − A)	0.09482	0.08745	0.09374
Chemical softness (S)	S = 1/ η	10.5463	11.4351	10.6684
Electrophilicity index (ω)	ω = 2 (μ^2^/η)	0.14726	0.17104	0.14185
Nucleophilicity index (N)	N = 1/ω	6.7909	5.8465	7.0495
Additional electronic charge	=−μ/η	−1.7624	−1.9778	−1.7397

**Table 7:** Docking scores (kcal/mol) of selected ligands against off-target isoforms CA I and CA II.

Isoforms	Compound	Docking Score (Kcal/mol)
CA II (Carbonic Anhydrase II)	Native ligand	−8.9
	ZINC613262012	−8.1
	ZINC427910039	−7.8
	DB00482	−8.4
CA I (Carbonic Anhydrase I)	Native ligand	−6.5
	ZINC613262012	−7.2
	ZINC427910039	−7.3
	DB00482	−7.6

## Data Availability

Data is provided within the manuscript or Supplementary Information Files.

## Supplementary Materials

The following supporting information can be downloaded at: https://www.mdpi.com/article/10.3390/ijms26178465/s1.
