# Supplementary material for: Computational Discovery of Selective Carbonic Anhydrase IX (CA IX) Inhibitors via Pharmacophore Modeling and Molecular Simulations for Cancer Therapy
Source: Int J Mol Sci. 2025 Aug 30;26(17):8465. doi: 10.3390/ijms26178465 (PMC12429665; doi:10.3390/ijms26178465)
Supplement: Supplementary file 1 [file ijms-26-08465-s001.zip › ijms-3773615-supplementary.pdf]

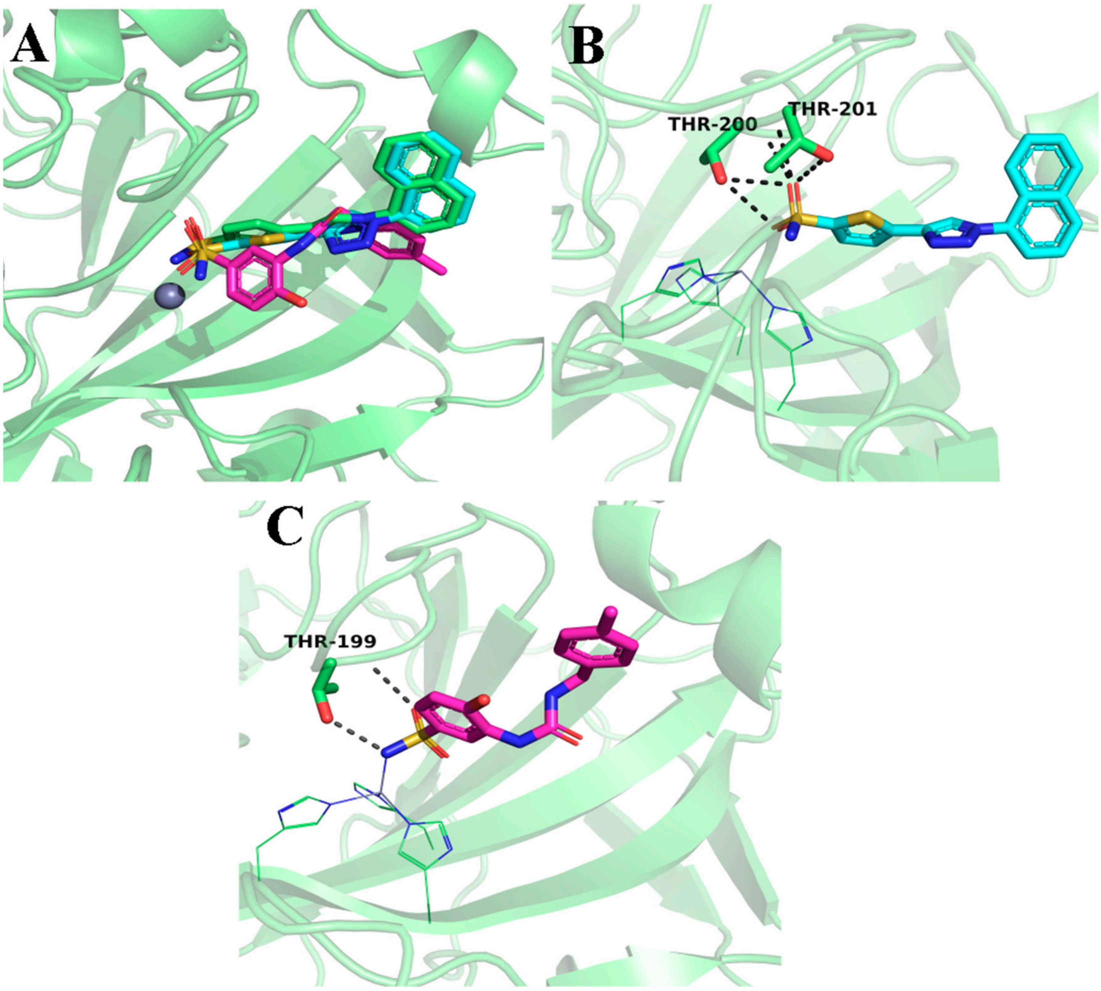

Figure S1 A) Re-docked conformation of the reference ligands 9FK (Blue), and SCL-0111(Pink) superimposed on the crystallized conformation of 9FK (Green). B) 9FK position and interactions within the active site. C) SCL-0111 position and interactions within the active site
